# My Dog Is Not My Cat: Owner Perception of the Personalities of Dogs and Cats Living in the Same Household

**DOI:** 10.3390/ani8060080

**Published:** 2018-05-24

**Authors:** Laura Menchetti, Silvia Calipari, Gabriella Guelfi, Alice Catanzaro, Silvana Diverio

**Affiliations:** Laboratory of Ethology and Animal Welfare (LEBA), Department of Veterinary Medicine, University of Perugia, via San Costanzo 4, 06126 Perugia, Italy; laura.menchetti7@gmail.com (L.M.); schatzi86@gmail.com (S.C.); gabriella.guelfi@unipg.it (G.G.); alice.catanzaro@libero.it (A.C.)

**Keywords:** pet-human relationship, dogs and cats, personality traits, five-factor model, genetic and environmental factors

## Abstract

**Simple Summary:**

A growing number of dogs and cats live together, sharing both a common home and common owner. Nevertheless, how do owners of both cats and dogs living in the same household perceive their pets’ personalities? We tried to answer this question by using a questionnaire targeted at people who owned both dogs and cats. Sociability, protectiveness, reactivity, neuroticism, and fearfulness were the traits that emerged and that diversified themselves according to species. Moreover, intrinsic animal factors, such as age and gender, demographic characteristics of the owner, and environmental context seem to modulate the perceived personality traits in a species-specific way. As personality might influence pets’ welfare and adaptability to the home environment, owners of multiple pets should know and take into consideration both common and differential aspects of their pet’s personality to optimise cohabitation among dogs and cats.

**Abstract:**

This study aims to define the personality traits perceived by the owners of multiple pets and to evaluate how they are modulated by experiential-environmental factors. A questionnaire was administered to 1270 owners of multiple pets (dogs and cats) to collect data on the demographics, management, and personality of their pets. Data were analysed by principal component analysis, bivariate, and multivariable models. Five personality traits emerged in dogs and cats: sociability, reactivity, protectiveness, neuroticism, and fearfulness. The owners perceived differences in the personality of their pet: dogs scored higher in sociability, protectiveness, and reactivity, while lower in the neuroticism dimension compared with cats (*p* < 0.001). Age similarly affected sociability (*p* < 0.01), and reactivity (*p* < 0.001) in both dogs and cats, while species-specific gender differences were found as to fearfulness (*p* < 0.05) and neuroticism (*p* < 0.001). The age of acquisition modulated several traits in dog personality, while living with conspecifics especially influenced cats. Physiological, behavioural, and evolutionary characteristics could explain species differences. Moreover, intrinsic and extrinsic factors modulated the five dimensions of dogs and cats in a diversified fashion, suggesting complex interactions between species and the environment. However, owners could have had different attitudes with their animals which could have influenced personality perception.

## 1. Introduction

Personality refers to underlying behavioural tendencies that differ across individuals, that are consistent within individuals over time, and that affect the behaviour that is expressed in different contexts [1,2]. The term personality, borrowed from psychology, is the starting point for research on animal personality [1,3]. Animal studies also benefit from the methodology of human research, often using factor-analytic approaches [3,4,5,6,7,8,9]. However, while in humans there is a considerable consensus in a five-factor model, called the Big Five, in animals a common framework for defining personality has not yet been developed, neither across nor within a species [3,4,5,9].

The lack of a conventional model is attributable not only to the dispersion of animal research across disciplines and testing instruments, but also to the intricate interactions between genes and environmental factors that influence personality traits [1,3,5]. Indeed, genetic and anatomical features could affect personality traits as any other phenotypic characteristic [1,4,5]. In addition, the environment in which animals were raised before expressing the behaviour, as well as the ecological and social niches encountered in their life, could be crucial factors shaping their personalities [1,2,4]. In the case of companion animals, the prevailing social niche may include its mother, its owner, and other intra- and inter-species animals living with them. Therefore, studies on pets’ personalities should consider the influence of all these genetic and environmental factors, as well as their interactions [10]. Finally, when the personality of a pet is assessed through the subjective owner’s judgment, it could be influenced by attitudes, attachment, or personality of the owner himself/herself [11,12,13,14,15].

The study of the multiple factors affecting animal personality could have significant implications for animal welfare. Personality traits influence the adaptation reactions to different environments, modulating physiological stress responses and, ultimately, changes in immunity [4,16]. An understanding of the factors involved in pets’ personalities may finally help in the optimisation of several aspects of their management, including physical and social contexts.

To date, the literature on pets’ personalities has been predominantly focused on dogs [6,17,18], while far less attention has been devoted to cats [7,19,20,21], although both species are fully adapted to the human environment, and both are considered as family members [17,22,23]. Traditionally, the dog has been reputed as “man’s best friend”, being domesticated more than 100,000 years ago [22,24]. The process of dog domestication has involved mainly behaviour and cognitive abilities allowing their involvement in many working and social contexts [25,26,27,28]. The cat domestication process seems more recent—ca. 9500 ago—and took a different trajectory [22,29]. Despite a shorter history of domestication, a natural selection process, and a more solitary existence, cats are appreciated by human beings because of their adaptability to small residences, independence, and ease of care [26]. The cat population kept indoors currently exceeds that of dogs both in Europe and in the United States [30,31]. Moreover, perhaps because of the partial overcoming of prejudices about their incompatibility or changes in human urban lifestyles, a growing number of owners choose to adopt both a dog and a cat [26,32]. However, there is a lack of comparative studies, and only a few of them have evaluated dogs and cats living under the same roof [4,23,26].

Taking into account this growing phenomenon, and comparing how environmental and social factors intervene in the personality of dogs and cats, we chose to administer a questionnaire to owners of both species. Theoretically, people owning either a dog or a cat may have a low preference for one of these species, thereby providing a more equitable judgment on their personality. One might expect parallels in personality traits of dogs and cats living in the same household, because they share the same social and physical environment [1,2]. Furthermore, if the influence of owner characteristics on the perceived personality of their pets is true [11,12,13,14,15], we do not expect differences between dogs and cats as they are judged by the same owner. Finally, although dog owner reports may have a higher potential for subjective bias compared to behavioural observations, owners have the opportunity to observe the animal in a variety of situations over an extended period making the questionnaire an appropriate measure of pets’ personalities [33].

The multiple factorial approach adopted in this study aims to define the personality traits perceived by the owners of dogs and cats living in the same household, as well as to evaluate how the experiential-environmental factors, and their interaction with the species, has modulated their personality traits.

## 2. Materials and Methods

### 2.1. Participants

A survey through a questionnaire was targeted at people who owned both dogs and cats. Data were collected over a three-month period (from May to July 2014) from 1270 residents in Italy, who voluntarily and anonymously completed the questionnaire. We could not calculate the relative response rate because the questionnaire was distributed through email and social networks, using the virtual snowball sampling technique.

### 2.2. Materials and Procedure

The study is part of a broader project, named “RandAgiamo^®^”, which aims to increase the adoptability of adult shelter dogs in the Umbria region of Italy (for details see [34]). The questionnaire was preceded by an introduction explaining the purpose of the study. Characteristics required to participate in the survey were: be 18 years or older and have owned a dog and a cat living in the same household; assurance that the survey would be anonymous; and instructions for completing. In particular, it was specified that if the participant had more than one dog or cat, the answers had to be referred to the first pet acquired.

Apart from the participant’s demographic characteristics, the questionnaire was specifically developed to collect data on three main areas concerning dogs and cats living in the same household: (a) owner-pet management choice and relationship; (b) personality traits of the dog and the cat; and (c) dog-cat relationship. This paper analyses only the area concerning pets’ personalities, which, in turn, is divided into three sections:

- Section A included five multiple-choice questions on participant demographic data: age, gender, region of residence, and number of pets owned. The respondent was also asked for his expertise on animals, then if he was a veterinarian, a dog trainer, a volunteer for animals, a dog/cat breeder, or an animal passionate.
- Section B consisted of 25 multiple-choice questions concerning the dogs’ physical characteristics (age, sex, de-sexing status, size, type), the amount of time it had been left with the mother and the age of acquisition, management (where the dog lives and sleeps), and personality of the owned dog. To facilitate comparison between dogs and cats, breed, age, the amount of time it had been left with the mother and age of acquisition were classified in categories as showed in Table S2. In order to define the dog’s personality, we used 15 descriptors selected from the bibliography [5,6,8,9,11,19,21] rated on a 6-point scale from 0 (*strongly disagree*) to 5 (*strongly agree*): playful; loving; sociable; shy; aggressive; calm; independent; lazy; fearful; patient; hyperactive; noisy; territorial; protective; and jealous.
- Section C contained the same questions as Section B, but about the cat.

### 2.3. Statistical Analysis

The data obtained from the questionnaires using Google Forms^®^ were entered into an Excel spreadsheet and transferred into the statistical program SPSS Statistics version 23 (IBM, SPSS Inc., Chicago, IL, USA) for analysis. The level of statistical significance was set at <0.05.

Distributions within categorical variables of owner and pet demographic characteristics were analysed using chi-square goodness of fit tests. We used the McNemar test and Pearson chi-square to test the independence of demographic characteristics of dogs and cats.

Behavioural descriptors of dogs and cats were preliminarily analysed by principal component analysis (PCA). Components (PCs) showing eigenvalues greater than 1 were retained and rotated with the Varimax method [10]. Only factor loadings with an absolute value greater than 0.4 were interpreted [10]. The items with cross-loading were eliminated. The scores of PCs were calculated by the normalisation (range [0,1]) of the weighted sum scores. Cronbach’s alpha assessed the reliability of the PCs.

Subscales of behaviours, as defined by the PCs, were analysed using paired *t*-tests to compare dogs and cats living in the same household. Then, we built multivariate models by the Generalized Linear Models procedure with identity link function and normal distribution. We stratified by species to show, in the easiest way, the interactions between species and environmental factors. Indeed, influence of the species was very strong, and the introduction of interaction terms into the models would have further complicated the presentation of the results. We included in these models the PCs as the dependent variable and all the factors (both related to the pet and the owner) as predictors. We used graphics tests to verify the assumptions and Bonferroni correction for pairwise comparisons. Estimated parameters (with standard error) and *p*-values from the Wald test were reported.

## 3. Results

### 3.1. Participants

The majority of participants were women (91.2%; *p* < 0.001) aged between 26 and 40 years (44.1%, *p* < 0.001). A large portion of participants were found to live in Northern (44.6%) or Central (31.5%) Italy (*p* < 0.001) and claimed to be an expert of both dogs and cats (65.6%; *p* < 0.001). Most participants owned one (56.4%) or 2–5 dogs (40.5%) and one (36.9%) or 2–5 cats (48.9%; *p* < 0.001). A higher number of participants owned only one dog rather than one cat, and 2–5 cats or more rather than 2–5 dogs or more (*p* < 0.001). Table S1 presents the participants’ demographic characteristics according to their gender.

### 3.2. Comparison of the Demographic Characteristics between Dogs and Cats

Demographic characteristics of the cat and the dog living in the same household are detailed in Table S2. Most of both the dogs and cats were older than two years, although cats were younger than dogs (*p* < 0.001). Female dogs (55.7%) were more represented than female cats (50.8%; *p* < 0.05). More than half of the participants (52.9%) had neutered both their cat and dog, but 36.1% had sterilised their cat but not their dog (*p* < 0.001). More than half of the participants owned both mixed-bred (51.6%), but purebred dogs (45.4%) were more represented than purebred cats (8.2%; *p* < 0.001). More cats had been left with their mother less than one month compared with dogs (33.7% and 19.6% in cats and dogs, respectively; *p* < 0.001) and were acquired before three months of age, compared with dogs (72.7% and 58.7% in cats and dogs, respectively; *p* < 0.001).

### 3.3. Dog and Cat Personalities

The PCA of the overall dataset revealed five components with eigenvalues greater than 1, accounting for 69% of the total variance (Table 1). The items ‘lazy’ and ‘patient’ were removed because the cross-loaded onto two components. The five factors appeared easily interpretable. PC1, named “sociability”, was most influenced by social traits, such as loving, sociable, and playful. PC2, named “protectiveness”, included protective, territorial, and jealous traits of the animal. PC3, named “reactivity”, indicated the degree to which the pet was calm-hyperactive, whereas PC4, named “neuroticism”, included aggressive, shy, and independent adjectives. Finally, PC5, named “fearfulness”, was only accounted by one item, fearful, and then rather weak.

**Figure 1:**
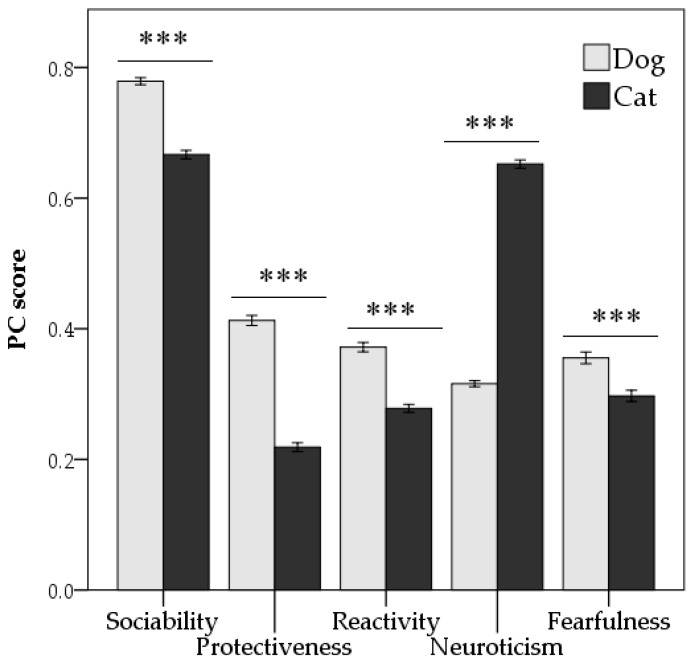
Comparison of personality traits, as extracted by principal component analysis, between dogs and cats living in the same household. Values are means ± standard error. *** *p* < 0.001 (paired *t*-test).

The five behavioural subscales, as resulted by scoring of PCs, were subsequently analysed by a paired *t*-test to check whether there were differences between dogs and cats living in the same household. Dogs scored higher in sociability (*p* < 0.001), protectiveness (*p* < 0.001), reactivity (*p* < 0.001), and fearfulness components compared with cats, that scored higher in neuroticism PCs (*p* < 0.001) compared with dogs (Figure 1).

### 3.4. Multivariate Models Determining What Factors Influence Dogs’ and Cats’ Personality

Both in dogs and in cats, sociability (Table 2) reduced with age. Dogs’ sociability was also negatively associated with age of acquisition (*p* < 0.001). Cat’s sociability was higher in males (*p* < 0.001), in North than Central or South Italy residents (*p* < 0.01) and increased as the number of cats in the house increases (*p* < 0.001).

Protectiveness of dogs reduced with age of acquisition (*p* < 0.01) and age of owner (*p* < 0.05). It was higher in dogs living indoors or both indoors and outdoors than only outdoors (*p* < 0.01) and was affected by sleeping habits (*p* < 0.05; Table 3). In cats, protectiveness increased if they remain for more than three months with its mother (*p* < 0.05) and was affected by living habits (*p* < 0.001; Table 3).

Reactivity reduced with the age of both dog and cat (*p* < 0.001) and with the age of acquisition (*p* < 0.05). It was also affected by age and expertise of owners in both dogs and cats (*p* < 0.05; Table 4). In particular, in dogs it reduced with the age of owner (*p* < 0.001). Reactivity was lower in purebred dogs (*p* < 0.05) and in dogs sleeping outdoors than indoors (*p* < 0.01). In cats, reactivity was affected by the region of residence (*p* < 0.01).

Neuroticism was higher in dogs and cats sleeping outdoors than indoors (*p* < 0.05; Table 5). In dogs was also affected by breed (*p* < 0.001) while in cats it was negatively associated with age (*p* < 0.01) but positively with age of owner (*p* < 0.01) and number of pets in the house (*p* < 0.001). Neuroticism was higher in male than in female cats (*p* < 0.001; Table 5).

Fearfulness of dogs was higher in females (*p* < 0.05), in mixed breed (*p* < 0.001), and in dogs living outdoors than indoors or both in and outdoors (*p* < 0.05). It was positively associated with age of acquisition (*p* < 0.01; Table 6). In both dogs and cats, fearfulness was negatively related with the age of owner (*p* < 0.05) and the number of conspecifics in the house (*p* < 0.01). Fearfulness was lower in cats sleeping outdoors than indoors (*p* < 0.05; Table 6).

## 4. Discussion

Five personality traits link dogs and cats: sociability, protectiveness, reactivity, neuroticism, and fearfulness. However, the owner recognises differences in the personality of his/her pets, assigning to dogs and cats living under the same roof species-specific personality traits. Moreover, many variables modulate the traits of dogs and cats in a diversified manner, suggesting complex interactions between genetic, environmental, and experiential factors. 

To the best of our knowledge, this is the first study which compares the personalities of both dogs and cats as perceived by a unique owner and emerged from a single PCA. This innovative approach allows us to refine the perception of the personalities of pets, reducing possible bias due to variances in the subjectivity of two different owners. The judgment of a unique owner who did not show a preference for dogs or cats could be freer from discrimination of species. Moreover, paired comparisons between species can be achieved using the same PCs, i.e., the same “units of measurement”. Finally, this method allows us to evaluate the effects on personality traits of species, environment (ontogenetic, management, and social), as well as the interaction between species and environment.

Factors emerging from our PCA have elements in common with other studies on humans and pets, but also have some innovative aspects. In particular, the sociability and neuroticism dimensions are personality traits also included in the five-factor model, the most widely accepted structure of human personality, and show considerable generality across species [3,4,8]. Conversely, reactivity, protectiveness, and fearfulness are additional dimensions to the five-factor model. Reactivity emerged in several studies on non-human animals [3,5,7,32,35], while protectiveness has been previously used in the description of dogs’ personalities [8], but did not emerge in studies evaluating cats [7,9]. In cats, instead, some authors have identified dimensions such as dominant and curious [36,37] or human aggressive [7]. Finally, fearfulness emerged as a separate component of our analysis, while often it is an item included in the neuroticism dimension [3]. However, our fearfulness PC included only one item and needs to be interpreted with caution.

A relevant finding for the present work was the species-related differences perceived by the owner. Disparities in scores obtained for each trait between the two species could be due to physiological and ethological species-specific characteristics, to different domestication processes, as well as to various human perceptions. Dogs have a long history of domestication—and then of commensalism—with humans, while others would argue that cats were never genuinely domesticated [22,26,38,39]. These different domestication processes could play a role in the evolution of their personalities, thus explaining why dogs scored higher in sociability, protectiveness and reactivity, while scoring lower in the neuroticism compared with cats. Our results are in keeping with those obtained by Serpell [11], who found dogs more playful, confident in unfamiliar situations, affectionate, active, friendlier, and less aggressive than cats.

However, we also hypothesised that prejudices and stereotypical perceptions could affect judgment on pets’ personalities. For example, owners more often describe their cat as independent as opposed to their dog [20]. Recently, O’Connor et al. [40] showed that dog adopters had higher expectations for their companion animal’s behaviour and human-pet relationships than cat adopters did.

Moreover, there are different human perceptions and knowledge of dog and cat personalities. First, behavioural traits of dogs are widely recognised [9,17,35], while cats’ personalities, behavioural needs, sociality, and cat-human communication are still poorly understood [9,19,36]. Cats may use different communication tools from dogs [20]. The latter may mislead the owner regarding the interpretation of behavioural traits of the two species. Cats may be more selective in social interactions, such as controlling the relationship with humans and may tend to look at the human less than dogs [19,20]. The owner could interpret these cat attitudes as a lack of sociability, opportunism, being timid, or independence.

After considerations of species differences, we performed a multiple regression to highlight experiential-environmental factors affecting pets’ personalities [10]. This multivariable model approach may be used in veterinary practice for predictive purposes. In fact, by using the regression algorithms, the veterinary behaviourist could obtain a score for each personality trait and identify critical points of pet management or facilitate the introduction of new animals in the household.

As previously shown [6,7,12,18,19], pets’ personalities were affected by gender. However, the gender did not seem to determine cross-species differences in perceived personality traits since, for example, male dogs were described as less fearful, while male cats scored higher in the sociability and neuroticism components than females. Accounting for potentially confounding variables, neutering did not affect any personality trait. Previous studies reported conflicting results with some reporting effects of neutering on several behaviours, such as calmness and destructiveness [6,12,17,18], otherwise no change [41,42]. As for cats, we cannot confirm previous studies claiming that neutering decreases aggressive behaviours [43,44]. Moreover, unlike the approach of prior studies, the possible effect of confounding variables on neutering, such as the age of the animal and the characteristics of the owner, always needs to be evaluated.

Comparisons between purebred and mixed dogs showed that purebred dogs are perceived as calmer, less neurotic, and more fearful. Several authors claimed that genetically-based differences in behavioural traits between purebred dogs [33,45] are probably linked to selection pressures for their traditional functions (i.e., working, sporting, hound, dog shows [46]). However, Svartberg [47] suggested that personality differences between breed groups were fleeting since dog domestication is still in progress. Our classification for purebred and mixed dogs, to make the two species comparable, did not allow us to make considerations related to breed genetic differences. In cats, there was no personality difference between purebred and mixed breed, as reported by Bennett et al. [36], but the low proportion of purebred cats could be a bias.

Personality dimensions are affected by genetics, as well as physiological factors, such as circulating hormone levels, hormone receptor density, and external factors [2,5]. Physiological changes, the environment encountered during different stages of life, and experiential factors, could contribute to the intraspecific differences related to age [1,2,4,5]. In this regard, we first note a considerable variability of pets’ scores under six months of age. This finding could suggest the lack of correspondence between behavioural characteristics of puppies and adults [35] and/or immaturity of personality traits in the young. The neuroendocrine networks and ecological and social niches have yet to play a role in personality traits of puppies/kittens, and this could explain their low temporal consistency [5]. Finally, in young animals, the owner, perhaps, did not have the time to identify a precise personality in his/her pet. Conversely, after the age of six months, we found clear age-related patterns of some personality traits crossing the two species: sociability and reactivity reduced both in dogs and cats. In addition to physiological changes, the length of ownership could help explain changes over time in the perception of pets’ personalities [13].

Surprisingly, the length of time spent with the mother did not affect many perceived behavioural traits of pets. Nonetheless, the role of maternal care up to the “socialisation period” of altricial species is well recognised. The mother is critical to the learning of social and adaptive behaviours, which also induces physiological and neurological changes [48,49,50]. Udell and Wynne [51] emphasise the role of the ontogenic environment on genes in the emergence of dog behaviour and some studies showed that the early environment of dogs affected subsequent social behaviour and coping styles [48,49]. The authors who studied the effect of early experiences on the subsequent personality of cats especially evaluated sociability, finding a match between the physical relationship of mother-offspring and the sociable behaviour in the adult, such as allogrooming or vertical-tail signal followed by head rubbing [52,53]. However, scientific literature in dogs and cats is still scarce compared to other species [48,49,50,52]. Our study may have a limit in this regard, as it used a retrospective questionnaire and participants may not remember, or do not know, information about the early life of their pet. The age at the time of acquisition mainly alters the dog personalities as defined by the owner. We can assume that later-adopted dogs have experienced social and spatial restrictions or other trauma during an early phase of their life, explaining the unfavourable scores for sociability, protectiveness, and fearfulness. Serpell and Jagoe [54] called the relationship between the age of acquisition and problem behaviours “kennel syndrome” and speculated that it could be due to the lack of early exposition to social and non-social stimuli.

In addition to experiences during the “socialisation period”, other environmental and social factors differently affect the perceived personality of dogs and cats, such as demographic characteristics and management choices of the owner. This finding suggests that complex interactions between environment and genotype modulate the personalities of dogs and cats, although they live in the same house and with the same owner. Furthermore, we also hypothesised that the owner’s attitudes could condition the perception of the pets’ personalities.

We found that younger owners enhanced the perceived protective instinct and dynamism of their dogs while dampening the neuroticism of their cats. Perhaps young owners have more free time and spend more time with their animals [13]. However, owners may engage in different activities with the two different pets, for example, mostly walking with dog and petting with cats, which may have different effects on their personality. Subsequently, even though dogs and cats share the same “family” setting, the owner may have different relational and managerial approaches with them. Therefore, as claimed by Bergmüller and Taborsky [2], the “shared environment” is often less important than the “non-shared environment” with respect to the personality development in animals. Owner expertise seems to influence reactivity, as reported in dogs by other authors [6,17]. Instead, unlike previous results [6,17], we did not find any relationship between owner gender and dog or cat personality. Finally yet importantly, the owner’s attitude could influence the behaviour and/or the interpretation of pets’ behaviour. For example, the older owner could perceive the dog as calmer and the cat as more neurotic because they are more experienced. Our survey did not include questions about personality or attitudes of the respondents. However, the effect of the region of residence on some PCs suggests that cultural factors of the owner may also influence the perception of the pets’ personalities [13]. Some recent reports on the dog-human relationship seem to confirm the link between owner and perceived or actual animal personality [12,13,14,15]. Cimarelli et al. [12] showed relationships between owner personality, interaction style, age, and dog behaviour. Sümegi et al. [15] hypothesized the presence of emotional contagion, Szaánthó [14] of empathy, while other authors [13,55] reported similarity or complementarity between the personality profiles of dogs and owners. Assuming that owner personality is influential, the perceived differences between species remain challenging to explain. Perhaps the owner creates different levels of empathy and/or different types of relationships that modulate the perception of animals’ personality. Further research on this topic would be of utmost importance.

Interestingly, living with conspecifics has essential importance on pets’ perceived personalities, especially as it regards the cat. Indeed, the presence of other cats improves the fear and sociability aspects but also increases the neurotic one. These results support the idea that the social life and interaction dynamics of the cat are quite complicated to understand! Some authors claim that socialisation in cats is a result of domestication since it is solitary in the wild [22,39,53] or that if early socialisation does not occur, cats may remain solitary throughout their life [22,56]. Moreover, several studies reported aggressive behaviours within artificially-constructed indoor colonies [57,58]. However, when there are sufficient food resources to support a group, both feral and free-living domestic cats can live within a colony, building affiliative or friendly relationships [7,53,59]. Therefore, cats can be housed in groups if they are well socialised, and there is sufficient space for feeding and elimination areas [56,59]. Our results support the hypothesis that cats are a social species since their sociability component increased in multi-cat households. Alternatively, the owner of many cats may have more opportunities to grasp their social traits. On the other hand, the increase in neuroticism suggests that the presence of conspecifics also intensifies stress-related reactions, probably due to competition for food, space or care. In dogs, only fearfulness was reduced when the number of other dogs in the house increased. In any case, the effects of multi-cat and multi-dog households should be considered, especially when introducing a new animal in the household. Indeed, personality can modulate the individual’s ability to cope with a challenging environment affecting the physiological stress response and welfare [4,16]. For example, the high neuroticism of cats living with conspecifics confirms that the introduction of a new cat in the house could compromise their welfare and should be done so with caution [53,56,58]. Future studies could evaluate if the coexistence of dogs and cats in the same house mutually changes their personality by comparing owners who own only one of the two species.

Finally, we investigated the effects of perceived personality traits on living and sleeping habits. Keeping dogs in the home reduced their fear, but increased the protective instinct. Exclusive cohabitation with humans may serve as a secure base for the dog in exploring the environment [60] but could also result in “hyper-attachment” to the owner, isolation from the outside world, boredom, or anthropomorphism. Consequently, these type of owner-pet relationships have been found to be associated with dogs’ behaviour problems, such as aggression toward people or separation-related disorders [60,61]. For example, Jagoe and Serpell [62] found that aggression is minor if the dog does not sleep in the owner’s bedroom, while Diverio and Tami [18] found that house-living was associated with fear of loud noises in Argentine Dogos. On the other hand, proximity may involve spending more significant time together and/or have a stronger attachment that could be linked to higher levels of trainability and sociability in the dog [6]. Finally, as discussed above, cohabitation may not change the pets’ personalities as much as its perception on behalf of the owner.

A similar effect could be found in cats since the sociability component increased and neuroticism reduced when cats sleep on the bed. Heidenberger [63] found that the bed is the favourite sleeping place of the cat providing a good, close relationship with its human companion. As for the dog, the attachment and the relationship with the owner may influence the cat’s personality [11]. However, it is difficult to establish the cause and effect relationship: is it human proximity that increases sociability or is it a more sociable cat which chooses to sleep in the bed? Again, could the owner’s attitude influence his managerial choices and his/her perception of the pet’s personality? By multivariate analysis, only protectiveness was shown to be affected by cat living habits, while most of the previous studies showed that keeping cats only indoors was associated with several behavioural problems [59,63]. Indeed, an indoor environment could be more impoverished and monotonous compared with an outdoor ones, and the lack of stimuli may result in boredom, anxiety, and stress [56]. Furthermore, typical behaviours, such as scratching objects and spraying urine, could be perceived as problematic by the owner when performed indoors [56,59]. However, our results did not highlight particular behaviour problems in cats living indoors only. Moreover, we need to take into account that having outdoor access may involve the cat in road accidents and it could become a member of a stray cat population [56]. Then, the issue of “cat indoors or outdoors” remains unresolved and, as suggested by Rochlitz [56], each situation should be assessed individually.

As regards to the limits of the present work, we can note the imbalance in the gender of participants, since nine out of ten were women, and the voluntary participation in the questionnaire [6,13,32]. However, the considerable number of participants supports the reliability of our results. Furthermore, some questions in the survey could be further pursued, such as the level of experience and attitudes of the respondents, as well as the source of the pet (i.e., breeders, shelter/rescue pets, or from family/friends). These may be subject to future research.

## 5. Conclusions

Our survey described differences in personalities of dogs and cats as perceived by their owners. We investigated the influences of species, intrinsic, and environmental factors, though we cannot exclude the impact of owner attitude in the perception of pet personality. Overall, five traits were extracted and labelled as ‘sociability’, ’protectiveness’, ‘reactivity’, ‘neuroticism’, and ‘fearfulness’. The sociability and neuroticism dimensions are personality traits also included in human five-factor models, while reactivity, protectiveness, and fearfulness are added ones. Inter- and intra-species differences were also investigated in these five factors.

Differences between the perceived personalities of cats and dogs living in the same household are attributable to physiological and ethological species-specific characteristics as well as to different domestication processes. We also hypothesised the influence of stereotypical perceptions of owners regarding cat and dog personalities, as well as inadequate knowledge of cat communication tools. Moreover, the owner could have built different types of relationships with dogs and cats that may have led to perceptions of diversified personalities. Indeed, in agreement with common belief, we found dogs to be more sociable and protective as opposed to cats, which were more neurotic. As expected, we discovered differences in personality traits linked to demographic features of pets. Some differences were cross-species, such as those relating to age, while the effects of gender differed among the species. Demographic characteristics, such as age, the region of residence, and management habits of the owner also influence the perceived dog and cat personality.

As pet personality might influence the adaptability to the home environment, both common and differential aspects identified in our work should be considered to optimise the coexistence of dogs and cats, or in the introduction of new animals in the home. Further studies could investigate the influence of owner personality and dog-cat or pet-owner relationship on personality traits, as well as any differences between single and multi-pet households.

## Figures and Tables

**Table 1:** Factor loadings for the behavioural subscales of dogs and cats living in the same household derived from a principal component analysis of survey items.

Item	Component				
	Sociability	Protectiveness	Reactivity	Neuroticism	Fearfulness
Loving	**0.861**	0.122	−0.069	−0.001	0.118
Sociable	**0.838**	−0.036	0.023	0.152	−0.236
Playful	**0.642**	0.148	0.444	0.020	−0.040
Protective	0.022	**0.806**	0.174	−0.070	0.003
Territorial	−0.009	**0.803**	0.095	0.016	−0.009
Jealous	0.155	**0.751**	0.081	−0.111	0.093
Hyperactive	0.227	0.161	**0.772**	0.026	−0.026
Calm	0.224	0.033	**−0.769**	0.177	0.064
Noisy	0.097	0.255	**0.670**	0.007	0.092
Aggressive	0.131	−0.091	0.064	**0.856**	−0.061
Shy	0.314	−0.202	−0.046	**0.733**	0.311
Independent	−0.235	0.072	−0.204	**0.634**	−0.235
Fearful	−0.109	0.085	−0.006	−0.047	**0.933**
% Variance explained	22.22	18.00	10.84	9.94	8.40
Cumulative % variance explained	69.40				
Cronbach’s alpha	0.689	0.729	0.620	0.631	-

Factor loadings with an absolute value greater than 0.4 are in bold.

**Table 2:** Multivariate regression analysis for sociability in dogs and cats living in the same house.

SOCIABILITY						
Predictor	DOG			CAT		
	B	SE	*p*	B	SE	*p*
AGE OF PET *	−0.04	0.008	**<0.001**	−0.05	0.063	**<0.001**
SEX OF PET						
Female vs. male	−0.01	0.013	0.538	−0.05	0.009	**<0.001**
NEUTERING STATUS						
Yes vs. no	−0.02	0.013	0.151	−0.03	0.025	0.280
BREED						
Purebred vs. mixed	−0.01	0.012	0.350	−0.04	0.026	0.170
AGE OF ACQUISITION *	−0.04	0.009	**<0.001**	0.02	0.012	0.097
AGE WITH MOTHER						
<1 week vs. >3 months	0.01	0.072	0.142	0.04	0.093	0.985
Until 1 month vs. >3 months	−0.05	0.064		0.03	0.090	
Until 3 months vs. >3 months	−0.01	0.063		0.04	0.090	
Unknown vs. >3 months	−0.02	0.064		0.03	0.090	
SEX OF RESPONDENT						
Women vs. men	0.01	0.020	0.592	−0.02	0.024	0.367
AGE OF RESPONDENT *	0.01	0.007	0.696	0.01	0.009	0.137
REGION						
Central vs. North	−0.01	0.013	0.682	−0.03	0.015	**<0.008**
South vs. North	−0.01	0.017		−0.06	0.020	
EXPERTISE						
Dog expertise vs. No expertise	−0.01	0.023	0.575	−0.03	0.028	0.073
Cat expertise vs. No expertise	−0.01	0.028		0.07	0.035	
Dog and cat expertise vs. No expertise	0.01	0.015		0.01	0.017	
N° OF DOGS/CATS *	−0.01	0.011	0.941	0.05	0.010	**<0.001**
LIVING HABITS OF PET						
Indoors vs. Outdoors	0.02	0.013	0.064	0.06	0.042	0.413
Both Indoors and Outdoors vs. Outdoors	0.05	0.025		0.05	0.040	
SLEEPING HABITS OF PET						
Enclosed space vs. Outdoors	−0.01	0.033	0.433	0.05	0.043	0.165
Home area vs. Outdoors	−0.01	0.029		−0.02	0.038	
Free in the home vs. Outdoors	0.02	0.027		0.01	0.035	
Bedroom vs. Outdoors	0.03	0.028		0.05	0.043	
On the bed vs. Outdoors	0.03	0.028		0.05	0.038	
Other vs. Outdoors	−0.01	0.056		0.03	0.044	

* included as continuous variable. Values in bold are statistically significant at an alpha of 0.05.

**Table 3:** Multivariate regression analysis for protectiveness in dogs and cats living in the same house.

PROTECTIVENESS						
Predictor	DOG			CAT		
	B	SE	*p*	B	SE	*p*
AGE OF PET *	−0.02	0.011	0.121	0.01	0.009	0.110
SEX OF PET						
Female vs. male	−0.02	0.017	0.346	0.02	0.014	0.177
NEUTERING STATUS						
Yes vs. no	−0.01	0.019	0.438	0.01	0.025	0.610
BREED						
Purebred vs. mixed	−0.03	0.017	0.053	0.01	0.027	0.985
AGE OF ACQUISITION *	−0.03	0.012	**0.005**	−0.01	0.013	0.532
AGE WITH MOTHER						
<1 week vs. >3 months	−0.09	0.099	0.411	−0.12	0.096	**0.024**
Until 1 month vs. >3 months	−0.06	0.088		−0.10	0.092	
Until 3 months vs. >3 months	−0.09	0.086		−0.15	0.092	
Unknown vs. >3 months	−0.09	0.087		−0.16	0.092	
SEX OF RESPONDENT						
Women vs. men	−0.04	0.028	0.114	−0.01	0.013	0.412
AGE OF RESPONDENT *	−0.03	0.010	**0.010**	−0.02	0.009	0.065
REGION						
Central vs. North	0.02	0.017	0.537	0.02	0.016	0.474
South vs. North	0.02	0.023		−0.01	0.020	
EXPERTISE						
Dog expertise vs. No expertise	−0.01	0.031	0.714	−0.06	0.028	0.121
Cat expertise vs. No expertise	0.01	0.039		0.02	0.036	
Dog and cat expertise vs. No expertise	0.02	0.020		−0.01	0.018	
N° OF DOGS/CATS *	−0.01	0.015	0.447	0.01	0.011	0.775
LIVING HABITS OF PET						
Indoors vs. Outdoors	0.06	0.017	**0.001**	−0.02	0.044	**<0.001**
Both Indoors and Outdoors vs. Outdoors	0.05	0.034		0.05	0.041	
SLEEPING HABITS OF PET						
Enclosed space vs. Outdoors	0.10	0.046	**0.014**	−0.01	0.044	0.789
Home area vs. Outdoors	−0.03	0.039		0.02	0.040	
Free in the home vs. Outdoors	−0.01	0.037		0.04	0.037	
Bedroom vs. Outdoors	0.04	0.038		0.05	0.044	
On the bed vs. Outdoors	0.05	0.039		0.05	0.040	
Other vs. Outdoors	0.02	0.077		0.04	0.045	

* included as continuous variable. Values in bold are statistically significant at an alpha of 0.05.

**Table 4:** Multivariate regression analysis for reactivity in dogs and cats living in the same house.

REACTIVITY						
Predictor	DOG			CAT		
	B	SE	*p*	B	SE	*p*
AGE OF PET *	−0.07	0.010	**<0.001**	−0.07	0.008	**<0.001**
SEX OF PET						
Female vs. male	−0.01	0.016	0.455	−0.01	0.013	0.680
NEUTERING STATUS						
Yes vs. no	−0.03	0.017	0.151	−0.03	0.023	0.166
BREED						
Purebred vs. mixed	−0.03	0.016	**0.037**	−0.02	0.024	0.365
AGE OF ACQUISITION *	−0.03	0.011	**0.004**	−0.03	0.011	**0.028**
AGE WITH MOTHER						
<1 week vs. >3 months	0.11	0.092	0.133	0.02	0.086	0.949
Until 1 month vs. >3 months	0.12	0.081		0.03	0.083	
Until 3 months vs. >3 months	0.08	0.080		0.02	0.082	
Unknown vs. >3 months	0.07	0.080		0.03	0.082	
SEX OF RESPONDENT						
Women vs. men	−0.04	0.026	0.165	−0.01	0.023	0.656
AGE OF RESPONDENT *	−0.03	0.009	**<0.001**	0.01	0.007	**0.029**
REGION						
Central vs. North	−0.01	0.016	0.476	−0.04	0.014	**0.009**
South vs. North	−0.03	0.021		−0.03	0.018	
EXPERTISE						
Dog expertise vs. No expertise	−0.05	0.029	**0.016**	−0.04	0.026	**0.046**
Cat expertise vs. No expertise	0.07	0.036		0.02	0.032	
Dog and cat expertise vs. No expertise	0.02	0.019		0.02	0.016	
N° OF DOGS/CATS *	0.01	0.014	0.873	−0.01	0.010	0.597
LIVING HABITS OF PET						
Indoors vs. Outdoors	−0.01	0.016	0.809	0.07	0.039	0.132
Both Indoors and Outdoors vs. Outdoors	0.02	0.031		0.05	0.037	
SLEEPING HABITS OF PET						
Enclosed space vs. Outdoors	0.12	0.041	**0.013**	−0.01	0.040	0.714
Home area vs. Outdoors	0.03	0.036		0.01	0.036	
Free in the home vs. Outdoors	0.01	0.034		−0.01	0.033	
Bedroom vs. Outdoors	0.03	0.035		0.02	0.040	
On the bed vs. Outdoors	0.05	0.036		0.01	0.036	
Other vs. Outdoors	−0.04	0.072		−0.04	0.040	

* included as continuous variable. Values in bold are statistically significant at an alpha of 0.05.

**Table 5:** Multivariate regression analysis for neuroticism in dogs and cats living in the same house.

NEUROTICISM						
Predictor	DOG			CAT		
	B	SE	*p*	B	SE	*p*
AGE OF PET *	0.01	0.007	0.496	−0.03	0.009	**0.001**
SEX OF PET						
Female vs. male	−0.01	0.011	0.520	−0.05	0.013	**<0.001**
NEUTERING STATUS						
Yes vs. no	0.02	0.011	0.116	−0.03	0.024	0.163
BREED						
Purebred vs. mixed	−0.04	0.011	**<0.001**	−0.03	0.026	0.204
AGE OF ACQUISITION *	0.01	0.008	0.339	0.02	0.012	0.079
AGE WITH MOTHER						
<1 week vs. >3 months	−0.04	0.061	0.546	−0.10	0.091	0.611
Until 1 month vs. >3 months	−0.05	0.054		−0.08	0.088	
Until 3 months vs. >3 months	−0.04	0.053		−0.06	0.087	
Unknown vs. >3 months	−0.06	0.054		−0.07	0.087	
SEX OF RESPONDENT						
Women vs. men	−0.02	0.017	0.380	−0.01	0.024	0.863
AGE OF RESPONDENT *	−0.01	0.006	0.396	0.02	0.008	**0.007**
REGION						
Central vs. North	−0.02	0.011	0.067	−0.03	0.015	0.058
South vs. North	−0.03	0.014		−0.04	0.020	
EXPERTISE						
Dog expertise vs. No expertise	0.02	0.019	0.511	−0.02	0.027	0.147
Cat expertise vs. No expertise	−0.02	0.024		0.06	0.035	
Dog and cat expertise vs. No expertise	0.01	0.013		−0.01	0.017	
N° OF DOGS/CATS *	−0.01	0.009	0.370	0.04	0.010	**<0.001**
LIVING HABITS OF PET						
Indoors vs. Outdoors	0.02	0.011	0.120	0.03	0.041	0.703
Both Indoors and Outdoors vs. Outdoors	0.04	0.021		0.03	0.039	
SLEEPING HABITS OF PET						
Enclosed space vs. Outdoors	−0.01	0.028	**0.039**	−0.05	0.042	**0.026**
Home area vs. Outdoors	−0.05	0.024		−0.12	0.038	
Free in the home vs. Outdoors	−0.04	0.023		−0.07	0.035	
Bedroom vs. Outdoors	−0.06	0.024		−0.06	0.042	
On the bed vs. Outdoors	−0.06	0.024		−0.09	0.038	
Other vs. Outdoors	−0.03	0.046		−0.02	0.043	

* included as continuous variable. Values in bold are statistically significant at an alpha of 0.05.

**Table 6:** Multivariate regression analysis for fearfulness in dogs and cats living in the same house.

FEARFULNESS						
Predictor	DOG			CAT		
	B	SE	*p*	B	SE	*p*
AGE OF PET *	−0.01	0.012	0.275	0.02	0.012	0.070
SEX OF PET						
Female vs. male	0.04	0.020	**0.034**	0.02	0.019	0.300
NEUTERING STATUS						
Yes vs. no	0.04	0.021	0.099	0.02	0.034	0.652
BREED						
Purebred vs. mixed	−0.07	0.020	**<0.001**	−0.05	0.035	0.132
AGE OF ACQUISITION *	0.05	0.014	**0.001**	−0.02	0.017	0.279
AGE WITH MOTHER						
<1 week vs. >3 months	0.07	0.115	0.828	0.09	0.127	0.119
Until 1 month vs. >3 months	0.10	0.102		0.16	0.122	
Until 3 months vs. >3 months	0.08	0.100		0.10	0.121	
Unknown vs. >3 months	0.07	0.101		0.12	0.121	
SEX OF RESPONDENT						
Women vs. men	−0.04	0.031	0.254	−0.02	0.033	0.496
AGE OF RESPONDENT *	−0.03	0.011	**0.015**	−0.03	0.009	**0.033**
REGION						
Central vs. North	0.01	0.020	0.452	0.04	0.021	0.179
South vs. North	−0.03	0.027		0.04	0.027	
EXPERTISE						
Dog expertise vs. No expertise	−0.05	0.036	0.336	−0.07	0.038	0.172
Cat expertise vs. No expertise	−0.05	0.045		0.01	0.047	
Dog and cat expertise vs. No expertise	−0.04	0.023		−0.01	0.024	
N° OF DOGS/CATS *	−0.05	0.017	**0.002**	−0.04	0.014	**0.003**
LIVING HABITS OF PET						
Indoors vs. Outdoors	−0.06	0.020	**0.017**	−0.08	0.058	0.250
Both Indoors and Outdoors vs. Outdoors	−0.06	0.039		−0.05	0.055	
SLEEPING HABITS OF PET						
Enclosed space vs. Outdoors	−0.03	0.052	0.376	0.17	0.059	**0.034**
Home area vs. Outdoors	0.05	0.045		0.09	0.052	
Free in the home vs. Outdoors	0.03	0.042		0.11	0.049	
Bedroom vs. Outdoors	−0.01	0.044		0.13	0.058	
On the bed vs. Outdoors	0.01	0.045		0.15	0.053	
Other vs. Outdoors	−0.03	0.087		0.06	0.060	

* included as continuous variable. Values in bold are statistically significant at an alpha of 0.05.

## Supplementary Materials

The following are available online at http://www.mdpi.com/2076-2615/8/6/80/s1, Table S1. Demographic characteristics of the participants. Table S2. Comparison of the demographic characteristics, age at the time of acquisition, living and sleeping habits of dogs and cats living in the same household.
