# Supplementary material for: My Dog Is Not My Cat: Owner Perception of the Personalities of Dogs and Cats Living in the Same Household
Source: Animals (Basel). 2018 May 24;8(6):80. doi: 10.3390/ani8060080 (PMC6025356; doi:10.3390/ani8060080)
Supplement: Supplementary file 1 [file animals-08-00080-s001.pdf]

Supplementary Material

Table S1. Demographic characteristics of the participants. Values are numbers and percentages (in brackets).

Characteristics		Women (*n* = 2314)	Men (*n* = 222)	Total
**Age of respondent (class)**	**18–25 years**	19 (17.1%)	198 (17.1%)	217 (17.1%)
	**26–40 years**	43 (38.7%)	516 (44.6%)	559 (44.1%)
	**41–55 years**	39 (35.1%)	360 (31.1%)	399 (31.5%)
	**56–70 years**	10 (9.0%)	83 (7.2%)	93 (7.3%)
**Region**	**North**	41 (37.3%)	513 (45.3%)	554 (44.6%)
	**Centre**	50 (45.5%)	426 (37.6%)	476 (38.3%)
	**South**	19 (17.3%)	193 (17.0%)	212 (17.1%)
**Expert**	**No expertise**	27 (24.5%)	222 (19.4%)	249 (19.9%)
	**Dogs expertise**	17 (15.5%)	102 (8.9%)	119 (9.5%)
	**Cats expertise**	3 (2.7%)	59 (5.2%)	62 (5.0%)
	**Dogs/Cats expertise**	63 (57.3%)	759 (66.5%)	822 (65.7%)
**N° dogs**	**1 dog**	62 (57.9%)	624 (56.3%)	686 (56.4%)
	**2–5 dogs**	43 (40.2%)	449 (40.5%)	492 (40.5%)
	**>5 dogs**	2 (1.9%)	36 (3.2%)	38 (3.1%)
**N° cats**	**1 cat**	48 (45.3%)	397 (36.0%)	445 (36.8%)
	**2–5 cats**	45 (42.5%)	547 (49.6%)	592 (49.0%)
	**>5 cats**	13 (12.3%)	159 (14.4%)	172 (14.2%)

Values are numbers and percentages (in brackets).

Table S2. Comparison of the demographic characteristics, age at the time of acquisition, and living and sleeping habits of dogs and cats living in the same household. Values are numbers and percentages (in brackets).

Parameter	Dog	Cat	*p* Value
**Age**			
**0–6 months**	23[a] (1.8%)	52[b] (4.1%)	0.0001
**6 months to 2 years**	264[a] (20.8%)	315[b] (24.9%)	
**2–8 years**	621[a] (49.0%)	561[b] (44.3%)	
**>4 years**	359[a] (28.3%)	338[a] (26.7%)	
**Sex**			
**Male**	560[a] (44.3%)	622[b] (49.2%)	0.013
**Female**	705[a] (55.7%)	643[b] (50.8%)	
**Reproductive status**			
**Entire**	545[a] (43.3%)	138[b] (10.9%)	0.0001
**Neutered**	715[a] (56.7%)	1130[b] (89.1%)	
**Breed**			
**Mixed***	664[a] (54.6%)	1060[b] (91.8%)	0.0001
**Purebred**	553[a] (45.4%)	95[b] (8.2%)	
**Age with mother**			
**<1 week**	31a (2.4%)	81[b] (6.4%)	0.0001
**Until 1 month**	219[a] (17.2%)	344[b] (27.3%)	
**Until 3 months**	572[a] (45.0%)	428[b] (34.0%)	
**>3 months**	10[a] (0.8%)	7[a] (0.6%)	
**Unknown**	438[a] (34.5%)	398[a] (31.6%)	
**Age at acquisition**			
**1–3 months**	736[a] (58.7%)	900[b] (72.7%)	0.0001
**4 months to 1 year**	309[a] (24.7%)	247[b] (20.0%)	
**1–8 years**	173[a] (13.8%)	85[b] (6.9%)	
**>8 years**	35[a] (2.8%)	6[b] (0.5%)	
**Where the pet lives**			
**Outdoors**	496[a] (39.4%)	69[b] (5.5%)	0.0001
**Indoors**	661[a] (52.5%)	588[b] (46.7%)	
**Outdoors and indoors**	103[a] (8.2%)	603[b] (47.9%)	
**Where the pet sleeps**			
**Free outdoors**	77[a] (6.1%)	95[a] (7.5%)	0.0001
**Enclosed space**	72[a] (5.7%)	53_{a} (4.2%)	
**Home area**	168[a] (13.3%)	163_{a} (12.9%)	
**Free in the home**	448[a] (35.4%)	599_{b} (47.5%)	
**Bed room**	253[a] (20.0%)	77[b] (6.1%)	
**On the bed**	232[a] (18.3%)	201[a] (15.9%)	
**Other**	16[a] (1.3%)	74[b] (5.9%)	

*including European cats. Values in the same row not sharing the same superscript are significantly different at $p < 0.05$.
